# A rapid evaluation of the *Rajiv Aarogyasri* community health insurance scheme in Andhra Pradesh, India

**DOI:** 10.1186/1753-6561-6-S1-O4

**Published:** 2012-01-16

**Authors:** Mala Rao, Shridhar Kadam, TN Sathyanarayana, Rahul Shidhaye, Rajan Shukla, Srikrishna Sulgodu Ramachandra, Souvik Bandyopadhyay, Anil Chandran, CT Anitha, M Sitamma, Mathew Sunil George, Vivek Singh, Subhashini Sivasankaran, Veena Shatrugna

**Affiliations:** 1 Indian Institute of Public Health, Hyderabad, India; 2 Indian Institute of Public Health, Bhubaneswar, India

## Introduction

In 2007, the Indian state of Andhra Pradesh introduced the *Rajiv Aarogyasri* community health insurance scheme (RAS) in order to break the vicious cycle of ill health, poverty, indebtedness and bankruptcy among families who are below the poverty line (BPL). The purpose of the scheme was to improve access to treatment of certain medical and surgical conditions for BPL families through a network of health care providers.

We conducted a rapid evaluation of RAS at the request of government of Andhra Pradesh. The purpose of the evaluation was to provide insights into the current performance of the scheme, to examine whether it is meeting the overall objectives and to suggest ways by which it may be further strengthened.

## Methods

We used secondary data on patients accessed through the trust that runs the scheme and conducted a survey in six randomly selected districts. Patient data were obtained from the Aarogyasri Healthcare Trust, which runs the programme. A total of 105,712 treatments had been authorised from April 1, 2007 to September 30, 2008. We analysed 89,699 treatments undertaken for 71,549 beneficiaries. We excluded 16,013 treatments for which data was not complete.

We conducted surveys in six randomly selected districts of the state. 217 beneficiaries from 18 *mandals* (administrative sub-division of districts) of six districts were interviewed at their homes. We visited nine *Andhra Pradesh Vaidya Vidhan Parishad* (APVVP) hospitals, four government teaching hospitals and 14 private hospitals. We also visited one primary health centre (PHC) from each *mandal*. We interviewed the stakeholders - state government, Aarogyasri Health Care Trust, Star Health Insurance Company and beneficiaries using semi-structured interview guides.

## Results

We found that 111 beneficiaries per 100,000 BPL population had utilised the scheme until the end of September 2008. Beneficiaries from the scheduled castes (SCs) and scheduled tribes (STs) were significantly lower than their proportions in the population, in a majority of the districts. Cardiac, cancer and neurological interventions made up 65% of all treatments administered by the scheme.

Of the 353 participating hospitals, 30 hospitals located in six cities of the state had undertaken more than 50% of all interventions. It was also observed that with increasing distance to major cities, the utilisation rate declined. The beneficiary satisfaction survey elicited the highest scores for doctors, nurses and cleanliness. The lowest scores were for health camps and information provided about the scheme.

Nearly 60% beneficiaries incurred a median out-of-pocket expenditure of INR 3600 (USD 77.3) with transport, medicine and pre-diagnostic investigations being the major reasons. 13% percent of beneficiaries had no follow-up visit and 28% had only one follow up visit.

## Discussion

The evaluation has revealed that there is scope for the scheme to improve strategic purchasing, quality of care, integration, continuous audit and in-built evaluation. The evaluation has emphasised on developing more coherent, cohesive and integrated health system with convergence of preventive, promotive and curative services taking into account the wider determinants of health.

